# Relational ontologies of AI and the technoethics of social isolation in psychosis

**DOI:** 10.1371/journal.pmen.0000508

**Published:** 2025-12-02

**Authors:** Hana Abbasian, Anil Fastenau

**Affiliations:** 1 Harvard Medical School, Boston, Massachusetts, United States of America; 2 Centre for Addiction and Mental Health, Toronto, Ontario, Canada; 3 Marie Adelaide Leprosy Center, Karachi, Pakistan; 4 German Leprosy and Tuberculosis Relief Association (DAHW), Wuerzburg, Germany; 5 Department of Global Health, Institute of Public Health and Nursing Research, University of Bremen, Bremen, Germany; 6 Heidelberg Institute of Global Health, University of Heidelberg, Heidelberg, Germany; PLOS: Public Library of Science, UNITED KINGDOM OF GREAT BRITAIN AND NORTHERN IRELAND

## Background

Artificial intelligence (AI) is increasingly implemented into mental health care, from predictive algorithms that flag relapse risk to digital companions offering cognitive-behavioral support [1]. Yet, most of these technologies are designed through an individualist lens, emphasizing symptom reduction, self-management, and cognitive correction [2]. What remains underexplored is how AI might support relational recovery, the process of re-establishing meaningful connections and belonging, particularly for individuals living with psychosis [3,4].

Relational ethics, an ethical framework that centers moral value in human relationships, mutuality, and interdependence, offers an important lens for reimagining the design and use of AI in mental health [5]. Relational ethics shifts the focus toward repairing the relational worlds disrupted by disorder, stigma, and social exclusion. This approach acknowledges that recovery is both internal and social. In this opinion piece, we call for a paradigmatic shift from individualist, symptom-oriented AI design toward relationally grounded systems that prioritize connection, recognition, and mutual care as central ethical and technological goals.

### Isolation as a moral and technological problem

Social isolation experienced by individuals living with psychosis can be understood as a structural and moral concern [6]. Experiences of stigma, epistemic marginalization, and the lack of supportive community networks contribute to what some scholars describe as a form of relational deprivation [6,7]. When technologies are developed without attentiveness to these ethical and contextual dimensions, they may unintentionally reproduce or worsen existing barriers to connection and care [6,8].

Multiple digital mental health apps focus on adherence metrics, such as how consistently a user engages with the tool for supporting users living with psychosis [8]. However, adherence-centered design can inadvertently frame non-use as moral failure, further alienating users who already feel marginalized by clinical and social systems [8,9]. A relation ethics approach, instead, asks: How might AI facilitate connection, dignity, and recognition rather than compliance and monitoring?

### From monitoring to mediating relationships

AI systems could move from surveillance (tracking behavior) to mediation (facilitating social meaning). For example, conversational agents designed through relation ethics could function less as therapists and more as relational bridges, helping users rehearse social interactions, coordinate with care networks and re-engage with community-based activities [9,10]. Such designs would draw from participatory AI, a design method where users co-create technologies, shaping algorithms according to their lived experiences, to make sure that people living with psychosis guide what “connection” means in their own terms [11,12].

Relational ethics also invites a reconsideration of agency [5]. For people with psychosis, agency is complicated, and it may fluctuate, fragment, or feel co-constituted with others [6,13]. Traditional AI frameworks that rely on consistent behavioral patterns or linear rationality may pathologize this fluidity.

### Relational justice and digital care infrastructures

Individuals living with psychosis often experience digital marginalization, data privacy concerns, and algorithmic profiling that reinforces stigma [8,14]. Relational justice (the equitable distribution of social and relational goods, such as recognition and belonging) requires that AI systems involve those whose perspectives have been historically silenced in data-driven innovation [12,15].

A relation ethics framework can thus function as a counter-narrative to technological solutionism, the belief that complex social issues can be neatly solved by technology alone [12,15]. This framework also insists that AI for psychosis care must be relationally accountable to patients, caregivers, and communities alike [12].

In this sense, relation ethics unsettles the extractive logics of mainstream AI design, which too often treats human experiences as datasets to be mined rather than stories to be held [5,12]. Relational framework reframes technological care as a co-constructed practice of epistemic humility and mutual recognition and reclaims agency for those at the margins of both psychiatry and computation [5,8].

### Toward a relational future in AI mental health

In a world where social disconnection has become an epidemic, technologies grounded in relational ethics and social connection can help understanding belonging for those living with psychosis. A relational turn in AI applications for mental health extends beyond ethics into epistemology, and how it can validate and nurture human experience. Relationally informed systems of AI would treat interactions, context, and reciprocity as data in themselves. For individuals living with psychosis, such systems could help understand relational safety and reconstitute trust in both human and digital worlds. Therefore, the moral promise of AI lies in its capacity to restore relations and put forward care, recognition, and reciprocity as priorities in design. We thus call for a fundamental reorientation of AI in mental health, from technologies that monitor isolated minds to those that mediate and nurture relationships, placing relational ethics at the core of future innovation in psychosis care.
